# Evolution of patients with squamous cell carcinoma of upper aerodigestive tract

**DOI:** 10.1590/S1516-31802003000400003

**Published:** 2003-07-01

**Authors:** Ali Amar, Sergio Altino Franzi, Abrão Rapoport

**Keywords:** Head and neck neoplasms, Metastases, Recurrence, Second tumors, Neoplasias de cabeça e pescoço, Metástase, Recidiva, Segundo tumor

## Abstract

**CONTEXT:**

Local and regional recurrences are frequent in patients with squamous cell carcinoma of the upper aerodigestive tract and early diagnosis is important for salvage treatment.

**OBJECTIVE:**

To identify the period of highest risk for the development of recurrences after surgical treatment of squamous cell carcinoma of the upper aerodigestive tract, in spite of radical therapy, in order to plan the follow-up for these patients.

**TYPE OF STUDY:**

Cross-sectional, descriptive.

**SETTING:**

Department of Head and Neck Surgery/ Otorhinolaryngology, Heliópolis Hospital (Hosphel), São Paulo, Brazil.

**PARTICIPANTS:**

A review was made of the hospital records of 889 patients with squamous cell carcinoma of the upper aerodigestive tract surgically treated between October 1977 and December 1996: 364 had oral cavity tumors, 107 had tumors of the oropharynx, 152 of the hypopharynx and 266, larynx tumors. The disease was stage I in 14 patients, stage II in 117, stage III in 352, stage IV in 397 and 9 patients were not staged.

**MAIN MEASUREMENTS:**

The interval between treatment and recurrence of disease was evaluated. The results were expressed as medians, quartiles (25% to 75%) and percentiles (10% to 90%). The annual incidence of recurrences and second tumors was calculated.

**RESULTS:**

Seventy-four percent of the recurrences were diagnosed within 18 months post-treatment. The local and regional recurrences and distant metastases showed medians of 270, 210 and 435 postoperative days respectively. The incidence of a second primary tumor varied from 2 to 3.1% a year.

**CONCLUSION:**

The majority of recurrences occurred within 18 months after the initial surgical treatment. The incidence of a second tumor remained stable after the first post-treatment year.

## RESUMO

**CONTEXTO:**

As recidivas locorregionais são freqüentes nos pacientes com carcinoma epidermóide das vias aerodigestivas superiores e o diagnóstico precoce é importante para o tratamento de resgate.

**OBJETIVO:**

Identificar os períodos de maior risco de recidiva após tratamento cirúrgico do carcinoma epidermóide das vias aerodigestivas superiores, para o planejamento do seguimento destes pacientes.

**TIPO DE ESTUDO:**

Transversal, descritivo.

**LOCAL:**

Departamento de Cirurgia de Cabeça e Pescoço e Otorrinolaringologia do Hospital Heliópolis (Hosphel) São Paulo, Brasil.

**PARTICIPANTES:**

Foram revisados os registros hospitalares de 889 pacientes com carcinoma epidermóide do trato aerodigestivo superior submetidos a tratamento cirúrgico loco-regional entre outubro de 1977 e dezembro de 1996, dos quais 364 eram de cavidade oral, 107 de orofaringe, 152 de hipofaringe e 266 de laringe. Quanto ao estádio, 14 apresentavam estádio I, 117 estádio II, 352 estádio III, 397 estádio IV e 9 não foram estadiados.

**VARIÁVEIS ESTUDADAS:**

Calculado o intervalo de tempo entre o tratamento inicial e o diagnóstico das recidivas locais, regionais e à distância. Os resultados foram expressos em medianas, quartis (25% e 75%) e percentis (10% e 90%). Foram calculadas as incidências anuais de recidiva e segundo tumor.

**RESULTADOS:**

74% das recidivas foram diagnosticadas nos primeiros 18 meses póstratamento. As recidivas locais, regionais e as metástases à distância mostraram medianas de 270, 210 e 435 dias respectivamente. A incidência de segundo tumor primário variou de 2 a 3,1% ao ano.

**CONCLUSÃO:**

A maioria das recidivas ocorreu até 18 meses após o tratamento cirúrgico inicial. A incidência do segundo tumor se mantém estável desde o primeiro ano pós-tratamento.

## INTRODUCTION

Malignant neoplasms of the mouth, pharynx and larynx constitute about 6% of all cancer deaths in Brazil.^1^ Recurrences are frequent in patients with squamous cell carcinomas of the upper aerodigestive tract, especially among those with advanced stage disease (stages III and IV).^2^ The follow-up period is defined arbitrarily, but very often covers the first 12 to 24 months, the period in which most recurrences are diagnosed. Evaluation of the results is indispensable, although some authors have argued against this approach, because most recurrences are preceded by symptoms and salvage treatment has poor results.^3^ The need for complementary treatment, usually radiotherapy, and the need for rehabilitation bring about a closer relationship between the patient and the medical team. The follow-up should consider the patient's requirements as well as the availability of resources within the health system, including bioethical aspects that guarantee benefit and minimal harm. The main incentive for performing follow-up is the possibility of early diagnosis of recurrence, which may represent the best opportunity for salvage treatment with curative intent, but such follow-up also has extreme relevance to the medical team for evaluation of the results.

The aim of this study was to identify the period of greatest risk of recurrence after surgical treatment of squamous cell carcinomas of the upper aerodigestive tract. In order to plan the follow-up for such patients in Brazil, where it is common to lose patients from post-therapy care, it is necessary to establish a rigid protocol, to benefit these patients with advanced head and neck squamous cell carcinoma in stages III and IV.

## METHODS

The charts of 943 patients with squamous cell carcinoma of the mouth, pharynx and larynx, treated at the Heliópolis Hospital between October 1977 and December 1996, were reviewed. All patients underwent surgical treatment of the primary tumor and neck dissection, with or without complementary radiotherapy. Fifty-four patients were excluded from the study due to death during the postoperative period (up to 30 days). Thus, 889 patients remained in the study, of whom 809 were male and 80 female. With regard to the primary site, there were 364 cases of oral cavity, 107 of oropharynx, 152 of hypopharynx and 266 of larynx tumors. With regard to staging, 14 patients showed stage I, 117 stage II, 352 stage III and 397 stage IV; in 9 patients the records did not allow clinical staging. The distribution of patients by primary site and stage is shown in Table 1.

**Table 1:** Stage of the tumor at diagnosis according to primary site

	I	II	III	IV	Not Established	TOTAL
Oral cavity	6	69	126	159	4	364
Oropharynx	3	13	38	53	0	107
Hypopharynx	3	4	51	93	1	152
Larynx	2	31	137	92	4	266
**Total**	**14**	**117**	**352**	**397**	**9**	**889**

An evaluation was made of the incidence of local, regional, distant and second tumor recurrence, and also the time interval between treatment and diagnosis of the respective recurrence. The diagnosis of second tumors had histological confirmation. The diagnosis of local and regional recurrences or distant metastases was based on physical and complementary examinations, without histological confirmation. A distance of more than 2 cm between the previous resection and the new lesion was necessary for a second tumor at the same anatomical site to be diagnosed. Among patients with more than one recurrence at the same anatomical site, only the first recurrence was considered. Neck recurrences were considered if they were not associated with local recurrence or a second tumor.

The results were expressed as medians, quartiles (25% and 75%) and percentiles (10% and 90%). The annual incidence of recurrences and second tumors was calculated. The differences observed in quantitative variables were evaluated via the Mann-Whitney test, and differences with an alpha level of less than 5% were accepted.

## RESULTS

Local recurrences were diagnosed in 154 patients (17%): 95 (10%) had isolated neck recurrences, 48 (5%) had distant metastases and 76 (8%) developed a second tumor.

**Figure 1:**
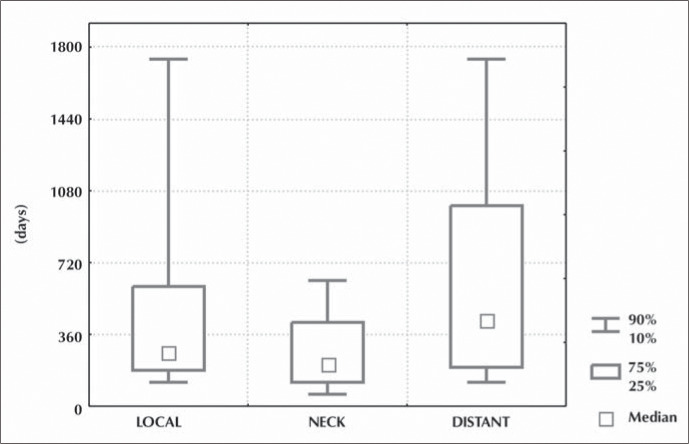
Time interval between initial treatment and local, neck and distant recurrences.

The diagnoses of local recurrences were made after a median of 270 postoperative days (range: 30 to 4,980), neck recurrences after a median of 210 days (60 to 2,010) and distant metastases after a median of 435 days (60 to 3,570) (Figure 1).

**Figure 2:**
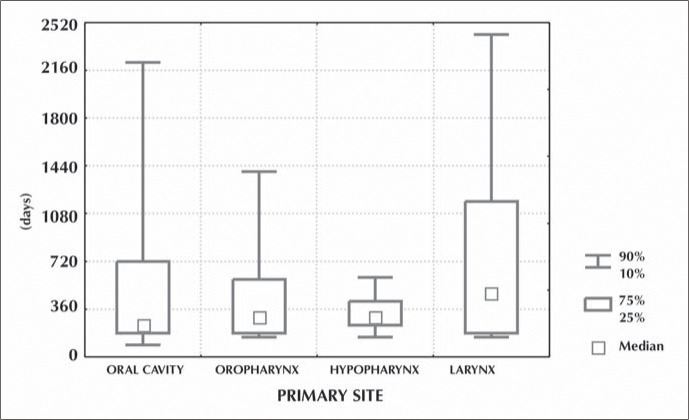
Time interval between initial treatment and local recurrence according to tumor primary site.

Local recurrences were diagnosed in 88 patients (24%) with oral cavity tumor, 21 (19%) in the oropharynx, 22 (14%) in the hypopharynx and 23 (8%) in the larynx. The larynx tumors had later local recurrences in comparison with the pharynx and oral cavity tumors, with medians of 480, 300 and 240 days respectively (Figure 2).

**Figure 3:**
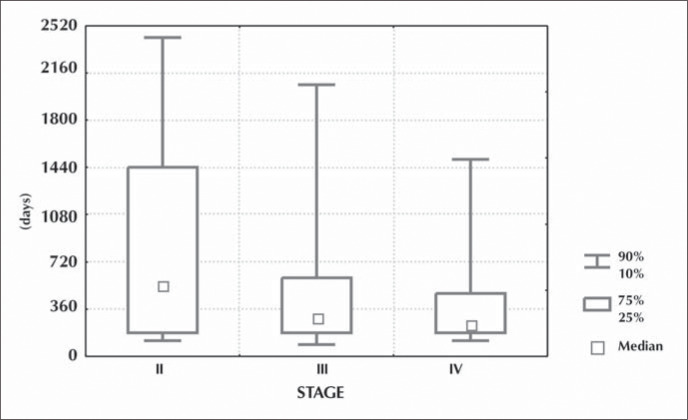
Time interval between initial treatment and local recurrence according to tumor stage at diagnosis.

Local recurrences occurred later in stage II tumors in comparison with those in stages III and IV, with medians of 540, 300 and 255 days respectively (Figure 3).

In patients who underwent postoperative radiotherapy, the local recurrences were diagnosed at a median of 300 days (Q_25-75%_ = 180-510) after the initial treatment, while in those who did not undergo postoperative radiotherapy, local recurrences were diagnosed at a median of 240 days (Q_25-75%_ = 180-810), p = 0.85.

Neck recurrences on the dissected side of the neck were at a median of 210 days (Q_2575%_=120-510), while the recurrences on the non-dissected side were at a median of 240 days (Q_25-75%_ = 120-450). The patients submitted to postoperative radiotherapy presented neck recurrences at a median of 285 days (Q_25-75%_ = 150-510) and the patients not submitted to postoperative radiotherapy had recurrences at a median of 180 days (Q_25-75%_ = 90-300, p = 0.03).

The incidence of a second tumor remained stable after the first post-treatment year, ranging from 2.0 to 3.1% per year.

**Figure 4:**
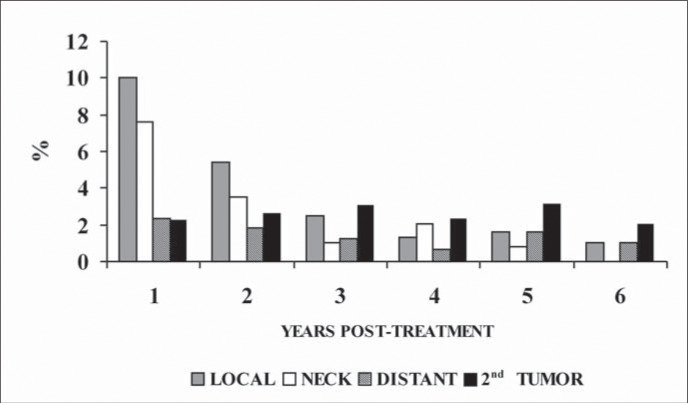
Incidence of local and neck recurrences, distant metastases and second tumors in years after beginning of treatment.

The local and regional recurrences occurred mainly within the first two post-treatment years (Figure 4). Of the 297 local and regional recurrences and distant metastases, 101 (34%) occurred within the first 6 months, 182 (61%) within 12 months, 220 (74%) within 18 months and 238 (80%) within the first 24 post-treatment months.

## DISCUSSION

The follow-up of patients with cancer of the upper aerodigestive tract has several objectives. Initially, these patients need rehabilitation with regard to phonetics and swallowing, which may include the use of prostheses. The high incidence of local and regional recurrences, especially during the first two years post-treatment, requires frequent follow-up with a view to the early diagnosis of these recurrences.^2,3^ Although up to 70% of the diagnoses of recurrences are preceded by symptoms, especially pain, in the other 30% of the cases the medical examination may detect a small lesion.^4^

Recurrence is a relevant prognostic factor: even among patients submitted to a new curative procedure, the disease control rates are low.^5,6^ Even though many of these patients are not able to undergo curative treatment when recurrence is diagnosed, there is a consensus regarding the maintaining of follow-up at frequent intervals during the first 12 to 24 post-treatment months. Schwartz et al.^5^ showed that the initial stage of the disease influences the prognosis more than the stage of the recurrence. Naturally, this fact reflects the difficulty of salvage surgery in a patient submitted to large-scale resection, and the initial stage shows more accurately the aggressiveness of the disease. It is not possible to state that early diagnosis of a recurrence provides a higher disease control rate, but this allows treatment with less morbidity. It can be comprehended that follow-up at frequent intervals has little impact on the survival rates. Nonetheless, the follow-up should consider ethical aspects so as to provide the best treatment.

Patients at an advanced stage of the disease constitute the majority within our society, and their treatment is a great challenge.^7^ Patients submitted to large-scale resections and postoperative radiotherapy are seldom salvaged after a recurrence, especially in early recurrences that are centered on the whole scar of the previous surgery. Early recurrences reflect inadequate therapeutic indication. Patients with recurrence during the first 6 months post-treatment do not benefit from therapeutic methods that have high morbidity and prolonged rehabilitation that takes up several weeks of their lives. According to Schwartz et al.,^5^ the patients who develop recurrences during the first 6 months have the worst results from salvage treatment. In the present study, one-third of the recurrences occurred within the first 6 months posttreatment.

The performing of follow-up is fundamental for the continuous evaluation of results: a difficult task when a significant number of patients are from a migrant population. Although survival is habitually expressed as actuarial curves of five or ten years, there is a trend towards decreasing the time interval for treatment evaluation. Mortality arising from other causes also becomes relevant in such a population, due to the innumerable comorbidities.^8^

Physiologically, there are two possibilities for recurrence: persistence of the tumor, which may remain quiescent for long periods, or a second lesion. A late recurrence seems to be more related to the patient's characteristics than to the effect of therapy and therefore, in carcinomas of the upper aerodigestive tract, the different therapeutic methods should be evaluated after an interval of two to three posttreatment years. As from the third year, the risk of a second tumor is equivalent to or overtakes the risk of local or regional recurrence. The criteria for the diagnosis of a second tumor, defined by Billroth in the 19^th^ century and Warren and Gates in 1932, are insufficient for accurately defining the nature of a late local recurrence, especially when we consider the field cancerization concept.^9-11^ In this context, the analysis of tumor genes may have more precise answers.

The neck recurrences in this study occurred later when patients were submitted to postoperative radiotherapy. This result cannot be attributed to the therapeutic effect alone, but also to the difficulty of early diagnosis of recurrences after irradiation of the neck. The radiotherapy had no effect on the timing of local recurrence, but these groups are not comparable: postoperative radiotherapy is generally recommended for patients with a higher risk of recurrence.

The distant metastases were diagnosed later, probably because complementary examinations were needed to establish diagnosis and fewer symptoms were caused. It is possible that distant metastases were underdiagnosed when they were synchronous with non-salvageable local or regional recurrences.

The incidence of second tumors was lower than has been found in other studies,^12^ possibly influenced by losses from follow-up and the higher disease stages in these patients. Monthly follow-up for long periods becomes impracticable for both the medical team and the patient. The patient should perform self-examination, pay attention to initial signs and symptoms and have easy access to specialized medical attention if he or she suspects any abnormality. This would allow follow-ups to be spaced at longer intervals (every six months or annually).

## CONCLUSION

Considering the high incidence of recurrences among patients with advanced cancer of the head and neck (stages III and IV), monthly follow-up during the first 18 months post-treatment, with the objective of early diagnosis of recurrences, is justified. The incidence of a second primary tumor, at a rate of around 2 to 3% a year, remains stable after the first year post-treatment and does not justify frequent follow-up. It is not necessary to make a gradual increase in the frequency of examinations during follow-up period in head and neck cancer.
